# *Clostridium difficile* Infections among Hospitalized Children, United States, 1997–2006

**DOI:** 10.3201/eid1610.100637

**Published:** 2010-10

**Authors:** Stephen M. Vindigni, Andi L. Shane

**Affiliations:** Author affiliation: Emory University School of Medicine, Atlanta, Georgia, USA

**Keywords:** Clostridium difficile, epidemiology, infection, bacteria, hospitalized children, diarrhea, letter

**To the Editor:** Zilberberg et al. described a notable increase in rates of *Clostridium difficile* infection (CDI)–related hospitalizations of children during 1997–2006 on the basis of analysis of data from 2 national administrative databases (*1*). As the authors acknowledge, they used administratively coded databases, which have inherent misclassification and testing biases.

Detection of *C*. *difficile* toxin indicates that bowel flora have been perturbed. However, the clinical role of toxin detection or isolation of *C. difficile* organisms in children is controversial. Although primary CDI is a recognized pathologic entity in children, one needs to consider whether another etiology related to a concomitant infection, antimicrobial drug administration, or alteration in enteral nutrition may be the precipitating event resulting in *C. difficile* toxin production.

It is our clinical observation that availability of testing for *C. difficile* and rapidity of assay results play a role in the submission of stool specimens for analysis. In 2007, we conducted a 5-month retrospective chart review of *C. difficile* testing practices at 2 local tertiary-care pediatric hospitals. Of 796 stool specimens submitted, 42 (5%) were notable for the detection of toxin A or B; these samples represented 35 patients (*2*). Medical coders likely face the same challenges as clinicians who must interpret toxin assay results and their clinical role with regard to hospitalized children. Although the ≈2-fold increase in CDI-associated hospitalization rates reported by Zilberberg et al. in their time series and cross-sectional analyses is notable, these results should be interpreted within the context of clinical and epidemiologic factors contributing to generation of this data.
