# Supplementary material for: Clostridium difficile Infections among Hospitalized Children, United States, 1997–2006
Source: Emerg Infect Dis. 2010 Oct;16(10):1651–2. doi: 10.3201/eid1610.100637 (PMC3294400; doi:10.3201/eid1610.100637)
Supplement: Related texts

# Reply: *Clostridium difficile* Infections among Hospitalized Children, United States, 1997–2006

**Authors:** Marya D. Zilberberg

**Affiliations:** Author affiliations: University of Massachusetts, Amherst, Massachusetts, USA;; Evi*Med* Research Group, LLC, Goshen, Massachusetts, USA

**Keywords:** Keywords: Clostridium difficile, epidemiology, infection, bacteria, hospitalized children, diarrhea, letter

**In Response:** I appreciate the letter by Vindigni and Shane pointing out the need for a cautious approach to treatment for infection with *Clostridium difficile* isolated from the stool of children, given their propensity for colonization by this organism (*1*). I could not agree more. In our report, we noted an increase over time in the rate of hospitalizations for not only *C*. *difficile* infections (CDIs) but also for rotavirus infections in children. This finding led us to acknowledge the possibility of a reporting bias for CDI (*2*). Other studies have detected a similar increase in CDIs among hospitalized children and have reported greater severity of associated disease (*3**–**5*). Such epidemiologic data, combined with emergence of the BI/NAP1/027 hypervirulent strain of *C*. *difficile* in the United States and abroad, support a real increase in CDIs in a population for which the clinical definition is likely less specific than for adults. Although a more precise clinical definition for CDI in children (primarily those <2 years of age) is needed, studies like ours, which are necessarily limited methodologically, can serve to alert clinicians to be more vigilant to the possibility of disease caused by this evolving pathogen, even in a population thought to be at low risk.

## References

[1] Vindigni SM, Shane AL. *Clostridium difficile* infections among hospitalized children, United States, 1997–2006. Emerg Infect Dis. 2010;16:1651.2087530910.3201/eid1610.100637PMC3294400
